# Plasma and urine metabolic profiles are reflective of altered beta-oxidation in non-diabetic obese subjects and patients with type 2 diabetes mellitus

**DOI:** 10.1186/1758-5996-6-129

**Published:** 2014-11-27

**Authors:** Jesús Zacarías Villarreal-Pérez, Jesús Zacarías Villarreal-Martínez, Fernando Javier Lavalle-González, María del Rosario Torres-Sepúlveda, Consuelo Ruiz-Herrera, Ricardo Martín Cerda-Flores, Erik Rubén Castillo-García, Irám Pablo Rodríguez-Sánchez, Laura Elia Martínez de Villarreal

**Affiliations:** Universidad Autónoma de Nuevo León, Hospital Universitario, “Dr. José Eleuterio González”, Servicio de Endocrinología, Monterrey, Nuevo León, 64460 México; Departamento de Medicina Interna, Universidad Autónoma de Nuevo León, Hospital Universitario, “Dr. José Eleuterio González”, Monterrey, Nuevo León 64460 México; Departamento de Genética, Universidad Autónoma de Nuevo León, Hospital Universitario, “Dr. José Eleuterio González”, Av. Gonzalitos s/n, Colonia Mitras Centro, Monterrey, Nuevo León 64460 México; Universidad Autónoma de Nuevo León, Facultad de Enfermería, Avenida Gonzalitos, 1500 Norte, Col. Mitras Centro, Monterrey, NL México

**Keywords:** Acetylcarnitine, Butyrylcarnitine, T2DM, Beta-oxidation defect, Non-diabetic obese, Air plethysmography

## Abstract

**Objectives:**

The two primary pathophysiological characteristics of patients with type 2 diabetes mellitus (T2DM) are insulin resistance (IR) and beta cell dysfunction. It has been proposed that the development of IR is secondary to the accumulation of triacylglycerols and fatty acids in the muscle and liver, which is in turn thought to be secondary to an enzymatic defect in mitochondrial beta-oxidation. The purpose of the present study was to analyze the molecules of intermediary metabolism to determine if an alteration in mitochondrial function exists in T2DM patients and, if so, to determine whether this alteration is caused by excess nutrients or an enzymatic defect.

**Design and methods:**

Seventy-seven subjects were recruited and divided into four groups (21 T2DM patients, 17 non-diabetic overweight/obese subjects, 20 offspring of T2DM patients, and 19 healthy subjects). Anthropometric parameters were determined by air plethysmography, and biochemical and metabolic parameters were measured, including 31 acylcarnitines (ACs) and 13 amino acids quantified by MS/MS and 67 organic acids measured by GC/MS.

**Results:**

Patients with T2DM showed elevation of short-chain ACs (C2, C4), a glycogenic amino acid (valine), a glycogenic and ketogenic amino acid (tyrosine), and a ketogenic amino acid (leucine) as well as altered excretion of dicarboxylic acids. T2DM offspring with abnormal glucose tolerance test GTT showed increased levels of C16. Subjects in the obese group who were dysglycemic also showed altered urinary excretion of dicarboxylic acids and lower levels of a long-chain AC (C14:2).

**Conclusions:**

These results suggest that mitochondrial beta-oxidation is altered in T2DM patients and that the alteration is most likely caused by nutrient overload through a different pathway from that observed in obese subjects.

## Introduction

Type 2 diabetes mellitus (T2DM) and obesity are two deleterious metabolic conditions [1] whose incidence has increased worldwide in the last decade [2]. Both diet and sedentary lifestyle are important risk factors for their development [3]. In Mexico, 74% of the adult population is either overweight or obese, and 14.6% suffer from T2DM [4], which has a high morbidity and mortality [2]. The primary pathophysiological characteristics in T2DM are insulin resistance (IR) and beta-cell dysfunction [5, 6]. IR is considered to be a state in which peripheral tissues are rendered unresponsive to the glucose lowering, antilypolytic, and anabolic properties of insulin, which is a hallmark of obesity and T2DM. IR is also accepted as an early feature of T2DM because it typically appears one or two decades before the manifestation of clinically overt diabetes [6].

Several studies suggest that IR occurs secondary to the accumulation of triacylglycerols and fatty acids in the muscle and liver (lipotoxicity theory) [6]. Although the molecular pathogenesis of lipotoxicity is not clear, it has been proposed that it occurs secondary to altered mitochondrial function, resulting from a decline in beta-oxidation, an excess of non-esterified fatty acids arriving at the mitochondria, or both [7]. Several reports indicate that there is an increased rate of beta-oxidation in obese individuals as well as in T2DM patients in a feeding state, whereas the rate of beta-oxidation is reduced during fasting [8]. Although the mechanisms of this reduction are unknown, it has been proposed that inhibition of carnitine palmitoyltransferase-1 (CPT1) as a result of increasing levels of malonyl coenzyme A (malonyl CoA) could be responsible for the decreased beta-oxidation activity [9]. Recent studies have suggested that lipid accumulation results from a lower oxidative capacity of the mitochondria [7] or reduced activity of the tricarboxylic acid cycle [6]. Another possible explanation for this reduction in beta-oxidation is an excessive increase in the delivery of fatty acids to the mitochondria [10].

Defects in the beta-oxidation of fatty acids can be evaluated based on acylcarnitine (AC) levels measured by tandem mass spectrometry, which is widely used in neonatal screening for fatty acid oxidation disorders and organic acidemias [11, 12].

This methodology has also been used to analyze mitochondrial function in diabetic patients. Recent reports indicate that there is an increase in the levels of long-, medium-, and short-chain ACs in the blood of patients with T2DM and an elevation of long-chain ACs in obese subjects [13].

We designed this study to quantify intermediate metabolites in plasma and urine to determine if they reflect a beta-oxidation defect in patients with T2DM and obese individuals and to determine whether an excess of nutrients or a blockage of beta-oxidation is the cause of the alteration. Additionally, we postulated that if there is an alteration in mitochondrial function in T2DM patients, it should be present before T2DM presents; for this reason, we explored beta-oxidation in the offspring of T2DM patients.

## Materials and methods

### Population

We performed a descriptive, comparative, non-blinded study that included 77 subjects (21 T2DM patients, 17 non-diabetic overweight/obese subjects, 20 non-diabetic offspring of T2DM patients [the latter two groups have a higher risk of developing T2DM], and 19 healthy subjects). Subjects with diabetes were recruited during 2010 from the outpatient diabetes clinic of the Endocrinology Service of the Dr. José E González University Hospital, Universidad Autónoma de Nuevo León (UANL), Monterrey, México. Volunteer participants were recruited from the University Hospital and Medical School population, either as subjects at risk for developing T2DM (overweight/obese and offspring of T2DM patients) or as controls. Written informed consent was obtained from all subjects, and the Health Research Ethics Board of the UANL Medical School approved the study (Approval #: EN-10-030).

Men and women over 18 years of age were included. Participants were assigned to one of four groups: 1) patients with T2DM diagnosed according to the criteria established by the American Diabetes Association; 2) non-diabetic subjects considered to be overweight/obese with a body mass index (BMI) ≥25 kg/m^2^ and without a history of T2DM in a first-degree relative; 3) non-diabetic individuals who had at least one parent diagnosed with T2DM; and 4) healthy individuals with a normal BMI (>20 and <25 kg/m^2^), a normal oral glucose tolerance test (OGTT), and no history of T2DM. The group of healthy subjects was designated as the control group. T2DM patients were required to discontinue medication (oral hypoglycemic drugs) and should not have received any insulin the night before the study.

### Anthropometric, biochemical, and metabolic parameters

Body composition was obtained by air impedance plethysmography (BOD POD). For the biochemical parameters, 30 mL of venous blood was collected after a 12- to 14-hour fast. Glucose levels, free fatty acids (FFAs), insulin, transaminases (AST, ALT), uric acid, urea nitrogen, cholesterol, and alkaline phosphatase as well as a lipid profile were examined in serum or plasma, depending on the kit used. The Homeostasis Model Assessment (HOMA) and Matsuda indices were calculated [14].

Blood samples were collected from all subjects to measure the levels of 31 ACs, 13 amino acids, pyruvate, lactate, and ketone bodies. An OGTT (standardized fasting) was performed in all groups except for the T2DM group. For AC quantification, blood samples were collected on filter paper (SS903) and analyzed by tandem mass spectrometry (MS/MS, API 2000, Perkin Elmer Sciex; full-scan, multiple-scan monitoring (MRM)).

A urine sample was collected for the measurement of 67 organic acids with a gas chromatograph/mass spectrometer (CLARUS 500, Perkin-Elmer Corporation, Norwalk, CT, USA). The procedure for the extraction of organic acids consisted of calculating the sample volume, then adjusting the volume according to creatinine excretion, which should be twice the volume containing 0.06 mg/100 g creatinine. Urinary organic acids were determined by oximation. Extraction was performed with ethyl acetate. Derivatization was performed by adding BSTFA-1% TMCS (N, O-bis (trimethylsilyl) trifluoroacetamide with 1% trimethylchlorosilane) and heating to 60°C in a water bath. Following this, the extract in solution was injected into the gas chromatograph. Finally, spectral analysis and identification were performed using the NIST MS Search Program Version 2.0.

### Statistical analysis

Each study group was compared with the control group. For quantitative parameters, Student´s *t*-test was used, whereas Fisher´s exact test was used for qualitative measurements. A *P* <0.05 was defined as significant. IBM SPSS 20 statistical software (IBM Corporation, Somers, NY) was used for data analysis.

## Results

In total, 111 biochemical and metabolic parameters, including ACs, amino acids, and organic acids, were measured in all groups. Comparison of the study groups with the control group showed that in the three groups of cases, the average BMI, anthropometric parameters obtained by BOD POD, and ages were higher.

Tables 1, 2, and 3 provide the biochemical parameters and metabolites that displayed significant differences between the case groups and the control group. Regarding biochemical parameters, subjects with T2DM had basal glucose, insulin levels, and ketone bodies levels in blood and a HOMA index that were significantly higher than those of healthy controls. Triacylglycerol, acetylcarnitine (C2), butyrylcarnitine (C4), alanine, tyrosine, and the branched chain amino acids leucine and valine (Table 1) were also significantly elevated in this group.

**Table 1:** **Comparison of anthropometric and biochemical measurements in T2DM patients and the control group**

	T2DM patients	Control (n = 19) (X + SD)	***P*** value
	(n = 21) (X + SD)		
**Age**	**52 ± 11.1**	**24.3 ± 3.7**	**<0.005**
**BMI**	**32.4 ± 6.0**	**22.8 ± 1.4**	**<0.005**
**Waist cm**	**103.3 ± 13.8**	**70.0 ± 7.7**	**<0.005**
**Hip cm**	**109.0 ± 13.3**	**95.0 ± 7.7**	**<0.005**
**% Fat**	**42.0 ± 8.9**	**25.6 ± 9.8**	**<0.005**
**% Lean mass**	**58.0 ± 9.0**	**74.3 ± 9.9**	**<0.005**
**Total weight**	**84.2 ± 17.1**	**63.1 ± 9.2**	**<0.005**
**Glucose 0´**	**148.2 ± 51.4**	**83.9 ± 12.5**	**<0.005**
**Glucose 30´**	ND	ND	ND
**Glucose 60´**	ND	ND	ND
**Glucose 90´**	ND	ND	ND
**Glucose 120´**	ND	ND	ND
**Insulin 0´**	**15.6 ± 7.9**	**7.3 ± 2.0**	**<0.005**
**HOMA IR**	**5.4 ± 2.7**	**1.5 ± 0.44**	**<0.005**
**Free fatty acids**	0.6 ± 0.2	0.5 ± 0.3	>0.05
**Beta-hydroxybutyrate**	0.2 ± 0.1	0.2 ± 0.04	>0.05
**ALP**	63 ± 17	62.10 ± 16.9	>0.05
**Uric acid**	5.8 ± 1.5	5.2 ± 1.0	>0.05
**Serum creatinine**	0.7 ± 0.17	0.8 ± 0.15	>0.05
**Cholesterol**	202.5 ± 50.7	200.6 ± 39.6	>0.05
**Total bilirubin**	0.2 ± 0.10	0.3 ± 0.2	>0.05
**Direct bilirubin**	.05 ± .02	0.06 ± 0.03	>0.05
**Indirect bilirubin**	0.15 ± 0.1	0.2 ± 0.1	>0.05
**Total protein**	8.1 ± 0 .7	8.5 ± 0.9	>0.05
**Albumin**	**4.7 ± 0.5**	**5.13 ± 0.5**	**<0.05**
**Globulin**	3.4 ± 0.4	3.4 ± 0.5	>0.05
**AST**	**17 ± 11.5**	**10.4 ± 2.0**	**<0.05**
**ALT**	**14.5 ± 10.1**	**8.0 ± 2.6**	**<0.005**
**C4**	**0.24 ± 0.10**	**0.18 + 0.08**	**<0.05**
**C2**	**10.1 ± 2.2**	**8.7 ± 1.6**	**<0.005**
**Leu**	**117.7 ± 22.3**	**96.1 ± 23.7**	**<0.05**
**Tyr**	**51.3 ± 10.4**	**41.5 ± 11.0**	**<0.005**
**Val**	**165.2 ± 19.0**	**135.0 ± 31.0**	**<0.005**
**Gly**	242.0 ± 36.2	226.5 ± 47.0	>0.05
**Arg**	27.5 ± 9.8	27.4 ± 7.8	>0.05
**Cit**	18.4 ± 4.7	17.5 ± 4.2	>0.05
**Met**	21.7 ± 5.1	22.4 ± 5.2	>0.05
**Orn**	83.7 ± 13.8	76.6 ± 9.6	>0.05
**Phe**	41.2 ± 6.2	39.3 ± 10.8	>0.05
**Ala**	**288.8 ± 63.0**	**238.0 ± 44.2**	**<0.005**

NOTE: Parameters in bold were significant.

**Table 2:** **Comparison of anthropometric and biochemical measurements in the overweight/obese patient group and the control group**

	Overweight/obesity	Control (n = 19) (X + SD)	***P*** value
	(n = 17) (X + SD)		
**Age**	**39.2 ± 14.0**	**24.3 ± 3.7**	**<0.005**
**BMI**	**30.2 ± 7.4**	**22.8 ± 1.4**	**<0.005**
**Waist cm**	**99.1 ± 17.6**	**70.0 ± 7.7**	**<0.005**
**Hip cm**	**111.3 ± 11.8**	**95.0 ± 7.7**	**<0.005**
**% Fat**	**37.3 ± 11.4**	**25.6 ± 9.8**	**<0.005**
**% Lean mass**	**62.7 ± 11.4**	**74.3 ± 9.9**	**<0.005**
	**30.6 ± 12.1**	**18.1 ± 11.7**	**<0.005**
**Total weight**	**84.1 ± 17.3**	**63.1 ± 9.2**	**<0.005**
**Glucose O´**	90.5 ± 11.9	83.9 ± 12.5	>0.05
**Glucose 30´**	144.2 ± 34.6	131 ± 30.9	>0.05
**Glucose 60´**	126.5 ± 53.5	113.9 ± 25.3	>0.05
**Glucose 90´**	120.5 ± 48.8	109.27 ± 33.33	>0.05
**Glucose 120´**	107.6 ± 46.4	94.9 ± 21.7	>0.05
**Insulin 0´**	10.7 ± 7.0	7.3 ± 2.0	>0.05
**Insulin 30´**	81.4 ± 51.5	56.7 ± 24.6	>0.05
**Insulin 60´**	89.9 ± 92.4	57.9 ± 36.2	>0.05
**Insulin 90´**	72.9 ± 63.3	54.8 ± 35.8	>0.05
**Insulin 120´**	76.3 ± 69.67	41.4 ± 37.0	>0.05
Matsuda index	4.9 ± 2.5	6.2 ± 1.8	>0.05
HOMA IR	2.3 ± 1.3	1.5 ± 0.44	<0.05
**Free fatty acids**	0.5 ± 0.20	0.5 ± 0.3	>0.05
**Beta-hydroxybutyrate**	0.2 ± 0.05	0.2 ± 0.3	>0.05
**ALP**	57.9 ± 13.1	62.16 + 16.9	>0.05
**Uric acid**	**6.3 ± 1.4**	**5.2 ± 1.0**	**<0.05**
**Serum creatinine**	0.9 ± 0.3	0.8 ± 0.15	>0.05
**Cholesterol**	214.0 ± 66.8	200.6 ± 39.6	>0.05
**Total bilirubin**	0.4 ± 0.3	0.3 ± 0.2	>0.05
**Direct bilirubin**	0.05 ± 0.03	0.06 ± 0.03	>0.05
**Indirect bilirubin**	0.3 ± 0.26	0.2 ± 0.18	>0.05
**Total protein**	8.2 ± 1.07	8.5 ± 0.9	>0.05
**Albumin**	4.9 ± 0.4	5.1 ± 0.5	>0.05
**Globulin**	3.3 ± 0.62	3.4 ± 0.5	>0.05
**AST**	**16.1 ± 9.3**	**10.4 ± 2.0**	**<0.05**
**ALT**	**10.4 ± 3.3**	**8.2 ± 2.6**	**<0.05**
**C4**	0.19 ± 0.11	0.18 ± 0.08	>0.05
**C2**	8.5 ± 2.3	8.7 ± 1.6	>0.05
**Leu**	95.3 ± 28.0	103.8 ± 21.5	>0.05
**Tyr**	43.4 ± 10.8	48.2 ± 10.5	>0.05
**Val**	126.1 ± 41.7	138.8 ± 34.2	>0.05
**Gly**	210.8 ± 54.1	220.6 ± 38.4	>0.05
**Arg**	24.3 ± 10.5	24.1 ± 6.25	>0.05
**Cit**	17.7 ± 4.1	17.9 ± 6.25	>0.05
**Met**	21.26 ± 5.7	23.5 ± 6.25	>0.05
**Orn**	85.5 ± 23.4	80.2 ± 15.0	>0.05
**Phe**	38.2 ± 8.5	41.1 ± 8.3	>0.05
**Ala**	209.9 ± 52.4	238. ± 45.2	>0.05

NOTE: Parameters in bold were significant.

**Table 3:** **Comparison of anthropometric and biochemical measurements in the T2DM patient offspring group and the control group**

	T2DM patient’s offspring	Control (n = 19) (X + SD)	P value
	(n = 20) (X + SD)		
**Age**	**37.15 ± 13.4**	**24.3 ± 3.7**	**<0.005**
**BMI**	**29.3 ± 6.0**	**22.8 ± 1.4**	**<0.001**
**Waist cm**	**95.3 ± 17.1**	**70.0 ± 7.7**	**<0.0005**
**Hip cm**	**106.0 + 17.7**	**95.0 ± 7.7**	**<0.005**
**% Fat**	**32.5 ± 11.2**	**25.6 ± 9.8**	**<0.05**
**% Lean mass**	**67.5 ± 11.2**	**74.3 ± 9.9**	**<0.05**
**Total weight**	**82.54 ± 21.67**	**63.1 ± 9.2**	**<0.0005**
**Glucose O**	90.25 ± 11.47	83.9 ± 12.5	>0.05
**Glucose 30´**	140.7 ± 32.76	131 ± 30.9	>0.05
**Glucose 60´**	**141.2 ± 40.0**	**113.9 ± 25.3**	**<0.05**
**Glucose 90´**	128.5 ± 46.46	109.27 ± 33.33	>0.05
**Glucose 120**	**120.05 ± 33.21**	**94.9 ± 21.7**	**<0.005**
**Insulin 0´**	**11.1 ± 4.9**	**7.3 ± 2.0**	**<0.0005**
**Insulin 30´**	**80.7 ± 49.8**	**56.7 ± 24.6**	**<0.05**
**Insulin 60´**	**87.7 ± 58.3**	**57.9 ± 36.2**	**<0.05**
**Insulin 90´**	**79.4 ± 57.2**	**54.8 ± 35.8**	**<0.05**
**Insulin 120´**	**73.2 ± 49.6**	**41.4 ± 37.0**	**<0.0005**
**Free fatty acids**	0.62 ± 0.24	0.5 ± 0.3	>0.05
**Beta-hydroxybutyrate**	0.18 ± 0.10	0.2 ± 0.3	>0.05
**ALP**	58.5 + 20.17	58.6 ± 20.2	>0.05
**Uric acid**	**6.21 ± 1.33**	**5.2 ± 1.0**	**<0.05**
**Serum creatinine**	0.91 ± 0.17	0.8 + 0.15	>0.05
**Cholesterol**	198.65 ± 28.4	200.6 ± 39.6	>0.05
**Total bilirubin**	0.34 ± 0.21	0.3 ± 0.2	>0.05
**Direct bilirubin**	0.05 ± 0.04	0.06 ± 0.03	>0.05
**Indirect bilirubin**	0.28 ± 0.21	0.2 ± 0.18	>0.05
**Total protein**	8.38 ± 0.80	8.4 ± 0.9	>0.05
**Albumin**	4.98 ± 0.39	5.11 ± 0.5	>0.05
**Globulin**	3.39 ± 0.55	3.4 ± 0.5	>0.05
**AST**	**14.85 ± 8.64**	**10.4 ± 2.0**	**<0.05**
**ALT**	**13.7 ± 8.11**	**8.2 ± 2.6**	**<0.0005**
**Triacylglycerol**	154.2 ± 114.9	110.6 ± 71.2	>0.05
**C4**	0.17 ± 0.08	0.18 ± 0.08	>0.05
**C2**	9.14 ± 1.44	8.7 ± 1.6	>0.05
**Leu**	103.8 ± 21.52	96.1 ± 23.7	>0.05
**Tyr**	48.2 ± 10.5	41.5 ± 11.0	>0.05
**Val**	138.8 ± 34.2	135.0 ± 31.0	>0.05
**Gly**	220.6 ± 38.4	226.5 ± 47	>0.05
**Arg**	24.1 ± 7.3	27.4 ± 7.84	>0.05
**Cit**	17.9 ± 3.09	17.5 ± 4.2	>0.05
**Met**	23.5 ± 6.25	22.4 ± 5.2	>0.05
**Orn**	80.2 ± 15.0	76.6 ± 9.5	>0.05
**Phe**	41.1 ± 8.3	39.3 ± 10.8	>0.05
**Ala**	231.0 ± 45.23	238.0 ± 44.2	>0.05

NOTE: Parameters in bold were significant.

In addition to differences in anthropometric measurements, non-diabetic overweight/obese subjects only had an increased HOMA index; we did not observe elevations of triacylglycerols, amino acids, or ACs (Table 2). In this group, six (35.3%) patients with pre-diabetes (basal glucose = 101 - 125 mg/dl and/or OGTT 120 min = 141 - 199 mg/dl) showed a decrease in the level of C14:2 (tetradecenoyl carnitine) in addition to glucose elevations at 30, 60, 90, and 120 min during the OGTT and elevation of insulin levels at 120 min compared with the control group (Table 4).

**Table 4:** **Comparison of anthropometric and biochemical measurements in the dysglycemic patient group and the control group**

	Dysglycemic/obese (n = 6 ) (X ± SD)	Dysglycemic/ offspring (n = 5) (X ± SD)	Control (n = 19) (X ± SD)
**Age**	53.0 ± 7.66*	46.6 ± 12.0*	24.3 ± 3.7
**BMI**	33.2 ± 8.28*	33.8 ± 6.7*	22.8 ± 1.4
**Waist cm**	99.9 ± 14.7*	109.0 ± 20.9*	70 ± 7.7
**Hip cm**	107.2 ± 8.7*	116.2 ± 24.5	95 ± 7.7
**% Lean mass**	66.8 ± 7.9	62.8 ± 15.1	74.3 ± 9.9
**Total weight**	87.2 ± 21.8*	95.8 ± 26.0*	63.1 ± 9.2
**Glucose O´**	97.2 ± 16.5	100.6 ± 8.8*	83.9 ± 12.5
**Glucose 30´**	169.7 ± 35.3*	177 ± 39.91	131 ± 30.9
**Glucose 60´**	182.8 ± 55.0*	196.2 ± 23.8*	113.9 ± 25.3
**Glucose 90´**	171.0 ± 42.8*	198.0 ± 38.5*	109.27 ± 33.33
**Glucose 120´**	150.0 ± 45.9*	165.0 ± 20.92*	94.9 ± 21.7
**Insulin 0´**	16.0 ± 9.6	12.6 ± 5.6	7.3 ± 2.0
**Insulin 30´**	87.4 ± 42.6	98.7 ± 54.3	56.7 ± 24.6
**Insulin 60´**	153.2 ± 135.6	126.2 ± 56.4*	57.9 ± 36.2
**Insulin 90´**	130.3 ± 77.3	131.7 ± 59.0*	54.8 ± 35.8
**Insulin 120´**	132.2 ± 84.1*	109.1 ± 46.0*	41.4 ± 37.0
**Matsuda index**	2.9 ± 2.1*	2.4 ± 1.0*	6.2 ± 1.8
**HOMA IR**	3.5 ± 1.5*	3.2 ± 1.5	1.5 ± 0.4
**Free fatty acids**	0.5 ± 0.3	0.7 ± 0.2	0.5 ± 0.3
**Uric Acid**	6.4 ± 1.4	7.12 ± 0.6*	5.2 ± 1.0
**Serum Creatinine**	0.9 ± 0.3	0.9 ± 0.2	0.8 ± 0.15
**Cholesterol**	234.5 ± 22.7*	180.8 ± 21.5	200.6 ± 39.6
**AST**	16.5 ± 7.6	18.2 ± 10.8	10.4 ± 2.0
**ALT**	12.0 ± 3.2*	19.4 ± 9.9	8.2 ± 2.6
**Triacylglycerol**	204.8 ± 80.0*	**283.6 ± 177.3**	110.6 ± 71.2
**C4**	0.18 ± 0.08	0.25 ± 0.13	0.18 ± 0.08
**C2**	8.4 ± 1.6	9.8 ± 1.05	8.7 ± 1.6
**C16**	0.74 ± 0.17	0.85 ± 0.14*	0.67 ± 0.17
**C 14:2**	0.03 ± 0.02*	0.05 ± 0.03	0.06 ± 0.3

*P value: <0.05.

NOTE: Parameters in bold were significant.

The non-diabetic offspring of T2DM patients showed higher blood glucose levels during the OGTT at 60 and 120 min as well as increased insulin levels at 0, 60, 90, and 120 min relative to the control group (Table 3). Additionally, the Matsuda and HOMA indices were lower and higher, respectively, as compared to control group (Table 3). In this group, 14 (66%) patients were overweight/obese, and 5 (25%) were pre-diabetic. The pre-diabetic offspring exhibited a significant elevation of palmitoylcarnitine (C16) and decreased glycine as well as significant differences in anthropometric measurements, insulin at time 0, and the HOMA index relative to the control group (Table 4).

All case groups demonstrated a significant elevation of transaminases (AST and ALT), although uric acid was only increased in the overweight/obese and offspring groups.

Analysis of the organic acids in urine showed the presence of intermediate metabolites of glycolysis and the Krebs cycle including lactic, 3OH-butyric, succinic, adipic, palmitic, citric, and phenyl acetic acids in all subjects in all four groups. Some metabolites were detected in a limited number of study participants. A significantly higher proportion of subjects with T2DM excreted 2OH-butyric acid relative to the control group (90% vs. 20%, *P* <0.05), and none exhibited sebacic acid excretion, compared with 40% in the control group (*P* <0.05). A lower number of obese subjects excreted suberic acid relative to the control group (36% vs. 89%, *P* <0.05). There were no differences in the excretion of organic acids between the offspring of T2DM patients and the control group.

## Discussion

The present study was conducted to obtain more information regarding biochemical and metabolic parameters in T2DM patients and to compare these parameters with healthy subjects to determine whether altered mitochondrial beta-oxidation, secondary to either an overload of nutrients or an enzymatic defect, exists in these patients. Additionally, the study examined whether subjects at risk for developing T2DM present metabolic alterations prior to developing the disease.

In the present study all the anthropometric parameters (i.e., BMI, waist, %fat) were significantly higher in the individuals of the case groups when comparing against the control group. However, only the offspring of T2DM patients showed altered plasma glucose and insulin levels during the OGTT; obese subjects did not.

In the biochemical measurements, transaminases (AST, ALT) were significantly higher in all case groups than in controls. It has been reported that individuals with T2DM have a higher incidence of liver function test abnormalities than individuals who do not have diabetes. Additionally, mild chronic elevations of transaminases often reflect underlying insulin resistance [15].

The elevation of transaminases in diabetics, overweight/obese individuals, and offspring of diabetic patients found in the present study may reflect fatty acid accumulation in the liver [16], although FFAs were not significantly elevated. It has been previously reported that elevated ALT in non-diabetic Swedish men is a risk factor for T2DM, independent of obesity, body fat distribution, plasma glucose, lipid, AST, bilirubin concentration, and family history of diabetes. In another study, non-diabetic Pima Indians were followed for an average of 6.9 years to determine whether hepatic enzyme elevations could be linked to the development of T2DM. At baseline, ALT, AST, and the OGTT were related to percent body fat. After adjusting for age, sex, body fat, whole body insulin sensitivity, and acute insulin response, only elevated ALT at baseline was associated with an increase in hepatic glucose output. Prospectively, increasing ALT concentrations were associated with a decline in hepatic insulin sensitivity and risk of T2DM. The authors concluded that higher ALT is a risk factor for T2DM and indicates a potential role of increased hepatic gluconeogenesis and/or inflammation in its pathogenesis [15]. Our results are in agreement with the aforementioned studies because only subjects with dysglycemia showed increased ALT in the subgroup analysis of the overweight/obese and offspring groups. As expected T2DM patients, showed high plasma triacylglycerol concentration, as well as disglycemic subjects from the obese and offspring groups.

It has been reported that an overload of mitochondrial lipid oxidation results in the accumulation of β-oxidation intermediates (ACs) and the depletion of Krebs cycle intermediates, leading to mitochondrial stress and activating currently unknown signaling pathways that interfere with insulin action [6]. Reports regarding the elevation of β-oxidation intermediates in diabetics are controversial. Shure et al. reported elevation of long-chain ACs and decreased levels of short-chain ACs [17], whereas Adams et al. and Mihalik et al. reported elevation of short-, medium- and long-chain ACs in diabetic patients [13, 18]. In a study with streptozotocin-induced diabetic rats, short-chain ACs (C2 and C4) was significantly elevated In a recent study, considerably higher levels of short-chain ACs and lower levels of some long-chain ACs were detected in T2DM patients and patients with metabolic syndrome [19].

In the present study, T2DM patients showed elevated levels of short-chain ACs (C2 and C4) in the blood as compared to healthy controls. Disglycemic offspring showed elevation of a long-chain AC (C16). Notably, in contrast to these groups, dysglycemic obese patients had lower levels of a specific long-chain AC (C14:2), which suggests the involvement of the same metabolic systems in a different manner, as postulated by Bene et al. 2013 [19].

Acetylcarnitine (C2) is the final metabolite of the beta-oxidation pathway, which produces acetyl CoA as a substrate for the Krebs cycle, whereas butyrylcarnitine (C4) is a marker of ketogenesis and mitochondrial beta-oxidation. C4 levels reflect the concentration of tissue butyryl CoA, which is a metabolite of glutamate and alpha-ketoglutarate, both of which are intermediate metabolites of the Krebs cycle [20]. These findings exclusively in the T2DM group would indicate an increase in mitochondrial beta-oxidation [21].

We believe that the differences in the levels of ACs found in the aforementioned studies, may be the result of the conditions or characteristics of the patients at the time of the study. In a previous study (unpublished results), we observed elevations of short-, medium-, and long-chain ACs in individuals with diabetes; however, in that study, patients had overt diabetes and were naive to treatment, which is in contrast to the patients in the present study, who were under treatment, and the differences among the reported studies could be related to the time of diagnosis, drug use, or BMI.

The AC profile is used in neonatal screening as an early marker of fatty acid disorders such as beta-oxidation enzymatic defects. The results of the present study do not support the hypothesis that there is an enzymatic defect in beta-oxidation that leads to fatty acid accumulation and precludes IR because the earlier manifestations of beta-oxidation overload were observed in the offspring of T2DM patients, who were already pre-diabetic. The pre-diabetic offspring showed significant elevation of long-chain fatty acid (C16) concentrations, whereas euglycemic offspring only showed a higher HOMA index relative to the control group, which suggests that some insulin resistance was already present. However, the elevation of C16 could be an early marker for the risk of developing diabetes, as previously reported by Zhao et al. [22].

T2DM encompasses not only changes in glucose metabolism but also alterations in fatty acid and protein levels with subsequent metabolic alterations in the pathways involved [23]. The branched-chain amino acids (BCAAs) leucine and valine are glycogenic and modulators of insulin secretion. According to Wang et al. [17], hyperaminoacidemia can promote diabetes via hyperinsulinemia.

In a previous report, Vannini P et al. reported that the BCAAs leucine and valine were increased in diabetic patients, indicating impaired short-term metabolic control [24].

BCAAs contribute to glucose recycling via the glucose-alanine cycle. Under normal conditions, alanine arising from BCAA nitrogen likely accounts for 25% of gluconeogenesis from amino acids [25, 26].

Elevation of BCAAs in IR adults independent of BMI in conjunction with increases in plasma ACs derived from amino acid oxidation suggests an increase in amino acid flux [27]. In a recent report, leucine, valine, tyrosine, and phenylalanine were found to be significantly associated with the incidence of diabetes [28]. In the present study, we observed a significant elevation of leucine, valine, tyrosine, and alanine in diabetic patients, although in contrast to the study of Wang, we did not find any association of the amino acid concentration pattern with the prediction of diabetes. Subjects in our study who were at risk for developing diabetes (overweight/obese and the offspring of T2DM subjects) did not show any increase in amino acid levels. In a study with diabetic db-/db- mice, clear evidence of increased gluconeogenesis was found, as demonstrated by strongly decreased concentrations of the gluconeogenic amino acids alanine, glycine, and serine [15]. Although in the present study we observed increased alanine in T2DM patients, dysglycemic obese subjects had lower levels of glycine, perhaps as a sign of altered glucose metabolism.

Obesity and a family history of T2DM are highly associated with development of the disease. The underlying mechanisms that trigger and exacerbate obesity-associated insulin resistance and the transition to T2DM remain unclear [29]. It has been suggested that a chronic positive energy balance and increased storage of energy as fat are linked to impaired glucose homeostasis and the development of diabetes [29]. Obesity is caused by the excessive accumulation of triacylglycerols, and its effects on the use and storage of various fuels (glucose, fatty acids, and amino acids) result in abnormalities in metabolism [30]. In the present study, several analytes such as transaminases, glucose, and insulin were elevated in both the obese group and the offspring group, which suggests that these metabolic alterations are already present in both groups. When we analyzed the pre-diabetic subjects, these metabolic abnormalities were present, and markers of altered mitochondrial beta-oxidation were also detected. We found a lower level of unsaturated long-chain fatty acids (C14:2) in the obese group, which could indicate a reduction in long-chain fatty acid metabolism. Increases in short-, medium-, and long-chain AC levels in diabetic individuals suggest a different and more complex defect compared with that of obese subjects.

Organic acids exist as intermediate compounds in many biochemical pathways, (glycolysis, glyconeogenesis, lipolysis). The Krebs cycle is the central metabolic pathway for energy molecules, and deficiencies in any of the Krebs cycle enzymes can cause inefficient cycling of organic acid intermediates, which consequently increase their concentration in the urine of the affected individual. In our study, differential urinary excretion of dicarboxylic acids was observed, whereas the excretion of adipic acid and 3OH-butyrate, which are indicators of ketogenesis, was similar in all groups. Ketosis is secondary to the fasting state and is a metabolic marker of fatty acid metabolism that is accompanied by excessive urinary excretion of adipic (C6) and suberic acids (C8) [31]. In the present study, a significantly lower proportion of obese subjects excreted suberic acid (C8) relative to the control group (36% vs. 89%), and none of the T2DM patients excreted sebacic acid (C10). This result could be related to a decrease in medium-chain fatty acids in T2DM patients, which has been reported previously [17].

The lack of sebacic acid (C10) excretion in diabetic subjects would indicate active lipid metabolism, as this has been reported in association with starvation, fat feeding, or experimental diabetes [32], as well as an elevated rate of beta-oxidation to form C6-C8 [33], contrary to what we found in dysglycemic obese patients.

The increased urinary excretion of hydroxyisobutyric acid in T2DM patients, which reflects an increase in serum C4 levels (derived from fatty acid oxidation or amino acid catabolism), indicates an increased metabolism of fats or proteins.

Specific patterns were observed for each analyzed group, and patients with T2DM had an abnormal AC pattern that suggests an increase in the substrate for mitochondrial beta-oxidation. This alteration could be present at earlier stages of the disease because pre-diabetic T2DM offspring showed increased levels of C16. The group of obese subjects also showed altered urinary excretion of dicarboxylic acids and lower levels of the long-chain AC C14:2 when pre-diabetic, suggesting altered mitochondrial beta-oxidation. Our results are in agreement with those reported by Koves, et al. (6), who suggested that there is a mitochondrial substrate overload in T2DM patients.

## Conclusion

Patients with T2DM exhibit defective beta-oxidation that suggests an overload of nutrients, as shown by higher levels of TGL and elevation of acetylcarnitine and butyrylcarnitine (both of which are derived from the final products of beta-oxidation) as well as augmented urinary excretion of intermediate metabolites. This beta-oxidation defect could be present at earlier stages of the disease because the pre-diabetic offspring of T2DM patients showed increased levels of C16. Moreover, obese subjects also showed altered mitochondrial beta-oxidation as well as altered urinary excretion of dicarboxylic acids and reduced levels of C14:2 only when disglycemic, indicating differential involvement of the metabolic pathways.
